# Qinggan Huoxue Recipe Alleviates Alcoholic Liver Injury by Suppressing Endoplasmic Reticulum Stress Through LXR-LPCAT3

**DOI:** 10.3389/fphar.2022.824185

**Published:** 2022-03-31

**Authors:** Yifei Lu, Mingmei Shao, Hongjiao Xiang, Junmin Wang, Guang Ji, Tao Wu

**Affiliations:** ^1^ Institute of Interdisciplinary Integrative Medicine Research, Shanghai University of Traditional Chinese Medicine, Shanghai, China; ^2^ Yueyang Hospital of Integrated Traditional Chinese and Western Medicine, Shanghai University of Traditional Chinese Medicine, Shanghai, China; ^3^ Teaching Department, Baoshan District Hospital of Intergrated Traditional Chinese and Western Medicine, Shanghai, China; ^4^ Institute of Digestive Disease, Longhua Hospital, Shanghai University of Traditional Chinese Medicine, Shanghai, China

**Keywords:** LXRα, LPCAT3, QGHXR, QGR, HXR, endoplasmic reticulum stress, alcohol liver injury

## Abstract

Endoplasmic reticulum stress (ERS) plays a key role in alcohol liver injury (ALI). Lysophosphatidylcholine acyltransferase 3 (LPCAT3) is a potential modifier of ERS. It was examined whether the protective effect of Qinggan Huoxue Recipe (QGHXR) against ALI was associated with LPCAT3 by suppressing ERS from *in vivo* and *in vitro* experiment. Male C57BL/6 mice were randomly divided into five groups (*n* = 10, each) and treated for 8 weeks as follows: the control diet-fed group (pair-fed), ethanol diet-fed group (EtOH-fed), QGHXR group (EtOH-fed + QGHXR), Qinggan recipe group (EtOH-fed + QGR), and Huoxue recipe group (EtOH-fed + HXR). QGHXR, QGR, and HXR groups attenuated liver injury mainly manifested in reducing serum ALT, AST, and liver TG and reducing the severity of liver cell necrosis and steatosis in ALI mouse models. QGHXR mainly inhibited the mRNA levels of *Lxrα*, *Perk*, *Eif2α*, and *Atf4* and activated the mRNA levels of *Lpcat3* and *Ire1α*, while inhibiting the protein levels of LPCAT3, eIF2α, IRE1α, and XBP1u and activating the protein levels of GRP78 to improve ALI. QGR was more inclined to improve ALI by inhibiting the mRNA levels of *Lxrα*, *Perk*, *Eif2α*, *Atif4*, and *Chop* and activating the mRNA levels of *Lpcat3* and *Ire1α* while inhibiting the protein levels of LPCAT3, PERK, eIF2α, IRE1α, and XBP1u. HXR was more inclined to improve ALI by inhibiting the mRNA levels of *Perk*, *Eif2α*, *Atf4*, and *Chop* mRNA while inhibiting the protein levels of LPCAT3, PERK, eIF2α, IRE1α, and XBP1u and activating the protein levels of GRP78. Ethanol (100 mM) was used to intervene HepG2 and AML12 to establish an ALI cell model and treated by QGHXR-, QGR-, and HXR-medicated serum (100 mg/L). QGHXR, QGR, and HXR groups mainly reduced the serum TG level and the expression of inflammatory factors such as IL-6 and TNF-α in the liver induced by ethanol. In AML12 cells, QGHXR and its disassembly mainly activated *Grp78* mRNA expression together with inhibiting *Lxrα*, *Lpcat3*, *Eif2α*, *Atf4*, and *Xbp1* mRNA expression. The protein expression of eIF2α and XBP1u was inhibited, and the expression of PERK and GRP78 was activated to alleviate ALI. In HepG2 cells, QGHXR mainly alleviated ALI by inhibiting the mRNA expression of *LPCAT3*, *CHOP*, *IRE1α*, *XBP1*, eIF2α, CHOP, and IRE1α protein. QGR was more inclined to inhibit the protein expression of PERK, and HXR was more likely to inhibit the protein expression of ATF4.

## Introduction

With the development of modern world, long-term and excessive drinking has become a global security problem, and it is closely related to many diseases, especially liver diseases (Menon et al., 2001; Im et al., 2021). They are all collectively referred to alcohol liver injury (ALI). The spectrum of development pathologically included mild alcoholic injury (MAI), alcoholic fatty liver (AFL), alcohol hepatitis (AH), alcoholic hepatic fibrosis (AHF), and alcoholic cirrhosis (AC). Epidemiology showed that 47.9% of all cirrhosis-related sclerosis patients died of AC (Rehm and Shield, 2013). The prevalence of ALI in China increased to 8.74% (Mathurin and Bataller, 2015; Chalasani et al., 2018; Wang et al., 2019) from 2000 to 2015 and showed a gradually increasing trend. In total, 90% of alcoholics develop AFL, while a small number of steatosis patients develop AH, and 10–20% eventually develops AC or even liver cancer (Mathurin and Bataller, 2015; Chalasani et al., 2018). ALI is one of the important processes in the development of liver diseases, mainly including the toxic effects of alcohol, acetaldehyde, lipid peroxidation, endotoxin, oxidative stress, and endoplasmic reticulum stress (ERS) (Suk et al., 2016), which straightly lead to the direct damage of the structure and function of the endoplasmic reticulum (ER) in hepatocytes (Howarth et al., 2012). When too many misfolded proteins accumulate in the ER, they stimulate downstream signaling pathways, mainly through the PKR-like ER kinase (*p*-PERK)/eucaryotic initiation factor 2α (eIF2α) pathway, inositol-requiring enzyme (IRE1)/X-box-binding protein 1 (XBP1) pathway, and activating the transcription factor 6 (ATF6) pathway. Early ERS is a cell self-protection mechanism, which can maintain cell homeostasis (Sugimoto et al., 2002; Singal and Shah, 2019), and long-term decompensated ERS can lead to steatosis, apoptosis, and inflammation (Longato et al., 2012). Our previous results confirmed the phosphorylation of eIF2α, and the expression of ERS molecular chaperone glucose-regulated protein 78 (GRP78) and apoptosis-related factor, caspase-3, was significantly downregulated in Qinggan Huoxue Recipe (QGHXR) (Han et al., 2011). Therefore, QGHXR is likely to alleviate ALI by regulating ERS; however, its mechanism needs to be further studied.

Lysophosphatidylcholine (lysoPCs), as the main component of cell membrane phospholipids (PLs), not only determines the biophysical properties of membrane but also affects the biological processes (Holzer et al., 2011), along with regulating immune cells, removing bacteria, and reducing neutrophils, tumor necrosis factor α (TNF α), and interleukin-1 β(IL-1β) (Drobnik et al., 2003; Yang et al., 2006). Stefanescu et al. (2016) found that serum lysoPC 16:1 and lysoPC 20:4 were specific biomarkers to predict the development of liver-related complications by observing 30 patients with ALD for 30 days. At the same time, our previous result showed that nine serum phospholipids decreased gradually with the progress of the disease from hepatitis, cirrhosis to liver cancer, especially lysoPCa C18:0, lysoPCa C18:2, lysoPCa C20:3, lysoPCa C20:4, lysoPCa C24:0, and PC ae C42:1 through targeted metabolomics (Wu et al., 2017). Our previous study first confirmed that LPCAT3 played a decisive role in the pathological changes of NASH (Xiang et al., 2021). All these results suggest that lysoPCs may be the key substance for predicting the occurrence and development of ALI. As another prevalent metabolic liver diseases globally, AFL also has shared and molecular regulatory mechanisms such as the SIRT1/AMPK (Berger et al., 2005; Chen et al., 2012) and PI3K/AKT signaling pathways (Zhang et al., 2021). Although the internal mechanism has not been fully clarified (Tarantino et al., 2019), further studies are required.

At present, clear and effective drugs for ALI are still lacking (Chalasani et al., 2018). Glucocorticoids, pentoxifylline, colchicine, insulin, and glucagon have been proved to have good curative effects in animals, but the clinical effect is not ideal (Singh et al., 2017). Traditional Chinese medicine (TCM) has stable efficacy in the treatment of ALI with fewer side effects such as gastrointestinal reactions and allergic reactions.

QGHXR includes five Chinese medicine including bupleurum, scutellaria, red sage, carapax trionycis, and radix puerariae. The main active ingredient of *Bupleurum* saikosaponin D has anti-inflammatory (Ma et al., 2016) and anticancer roles (Li et al., 2017). *Scutellaria baicalensis* flavonoids, the main active ingredient of scutellaria, can reduce liver injury caused by oxidative stress (Jia et al., 2021). Salvianolic acid A, the main active ingredient of red sage, can reduce the liver injury induced by concanavalin by activating SIRT1 and downregulating the p66shc pathway (Xu et al., 2013). Puerarin, the active ingredient of radix puerariae, can prevent the activation of pro-inflammatory factors and reduce lipopolysaccharide (LPS)/d-galactose (D-Gal)-induced liver injury by enhancing the ZEB2 expression level (Yang et al., 2021).

Our previous results had already confirmed that QGHXR not only significantly improved the clinical symptoms of ALI patients with total effective rate 95% but also reversed pathologic manifestations of hepatic steatosis and fibrosis (Gao and Shi, 2015). Animal experiments had also confirmed that QGHXR can inhibit liver lipid peroxidation damage caused by ethanol with anti-inflammatory and anti-hepatic fibrosis effects (Fu et al., 2005). *In vitro*, our previous result confirmed that QGHXR may inhibit CD14 and TLR4 by acting on the expression of nuclear factors NF-κB and AP-1 to protect hepatocytes (Wu et al., 2014), and QGHXR may inhibit epithelial–mesenchymal transition (EMT) by regulating the TGF-β1/Smads/Snail signaling pathway to alleviate AHF (Wu et al., 2016).

In order to study the effective parts of QGHXR for alleviating ALI, this study also analyzed the Qinggan Recipe (QGR) and Huoxue Recipe (HXR) according to their different roles. The present study is aimed to find the possible mechanism that QGHXR ameliorates ALI by inhibiting ERS through the LXRα-LPCAT3 signaling pathway both *in vivo* and *in vitro*.

## Materials and Methods

### Reagents

QGHXR consists of bupleurum (Jiangyin Tianjiang Pharmaceutical Co., Ltd., No. 19102524), scutellaria (Jiangyin Tianjiang Pharmaceutical Co., Ltd., No. 19070598), red sage (Jiangyin Tianjiang Pharmaceutical Co., Ltd., No. 19101349), carapax trionycis (Jiangyin Tianjiang Pharmaceutical Co., Ltd., No. 19090910), and radix puerariae (Jiangyin Tianjiang Pharmaceutical Co., Ltd., No. 19091622). QGR consists of bupleurum (Jiangyin Tianjiang Pharmaceutical Co., Ltd., No. 19102524) and scutellaria (Jiangyin Tianjiang Pharmaceutical Co., Ltd., No. 19070598), and HXR consists of red sage (Jiangyin Tianjiang Pharmaceutical Co., Ltd., No. 19101349), carapax trionycis (Jiangyin Tianjiang Pharmaceutical Co., Ltd., No. 19090910), and radix puerariae (Jiangyin Tianjiang Pharmaceutical Co., Ltd., No. 19091622). They were concentrated and dissolved to 7.41, 2.34, and 5.07 g/kg by using double distilled water, respectively. The contents of puerarin, baicalin, baicalein, and wogonin were processed though high-performance liquid chromatography to ensure the quality control of herbal medicine based on our previous reported study (Lu et al., 2010).

### Mice

Specific-pathogen-free 8-week-old male C57BL/6 J mice were provided by Shanghai SLAC Experimental Animal Co., Ltd. China and kept in the Animal Center of Shanghai University of Traditional Chinese Medicine at constant humidity (60 ± 5%), temperature (24 ± 1°C), and a 12 h light/dark cycle (6AM to 6PM light). The experiments were approved by the Guidelines for Animal Experiment of Shanghai University of Traditional Chinese Medicine, and the protocol was approved by the Institutional Animal Ethics Committee (PZSHUTCM200110003).

### Experimental Design

According to the formulation of the Gao–Binge–Lieber–Decarli liquid diet (Bertola et al., 2013a), after adaptation to isocaloric liquid diet (Bio-Serv, product no. F1259SP) for a period of 5 days, all mice were randomly divided into two groups as follows: 1) pair-fed mice (*n* = 10) that received only the isocaloric liquid diet; 2) EtOH-fed mice (*n* = 40) that received isocaloric and ethanol-containing liquid diet (Bio-Serv, product no. F1258SP) from Day 6 to Day 61. From Day 6 to Day 61, the EtOH-fed mice were divided into four groups: EtOH-fed + QGHXR (*n* = 10), EtOH-fed + QGR (*n* = 10), EtOH-fed + HXR (*n* = 10), and the EtOH-fed mice group (*n* = 10). The pair-fed group and EtOH-fed mice group were administered with 10 ml/kg saline per day; the QGHXR group received 7.41 g/kg, the QGR group received 2.34 g/kg, and the HXR group received 5.07 g/kg, respectively. EtOH-fed mice were gavage 31.5% (vol/vol) ethanol solution (5 g ethanol per kg of body weight) on Day 62 in the morning just before administration between 7:00 am and 9:00 am and 45% (wt/vol) maltose dextrin solution (9 g of maltose dextrin per kg of body weight) to the pair-fed group. All mice were anesthetized with 1% pentobarbital sodium at the dosage of 50 mg/kg and sacrificed 9 h later. The blood samples were then centrifuged at 4°C at 3,000×g, and the supernatant were collected for further analysis. In addition, 1 × 1 cm liver tissue was fixed for the histopathology analysis; some liver tissue was used for the quantitative real-time polymerase chain reaction (qRT-PCR) and western blot analysis (WB), and the remaining liver tissue was stored at −80°C.

### Biochemical Analysis

The serum level of alanine transaminase (ALT), aspartate transaminase (AST), triglyceride (TG), total cholesterol (TC) level, and hepatic TG and TC were analyzed using an automatic biochemical analyzer (Toshiba Accute Biochemical Analyzer TBA 40FR, Toshiba Medical Instruments, Otawara-shi, Tochigi-ken, Japan).

### Liver Histological Analysis

For analysis, 5-μm liver tissue section fixed in 4% paraformaldehyde was processed on slides for hematoxylin and eosin (HE) staining and observed under a light microscope (Olympus BX41TF, Olympus Corporation, Tokyo, Japan). Frozen tissue sections were analyzed by oil red O staining (CRYOSTAR NX50, Thermo Fisher Scientific, United States).

### Cell Culture and Treatment Including Collection and Production of Medicated Serum

HepG2 and AML12 cells were cultured in Dulbecco’s modified Eagle’s medium (DMEM) (Solarbio company, Beijing, China) (containing 10% fetal bovine serum, 100 U/ml penicillin, 100 μg/ml streptomycin, and 1‰ amphotericin B) and was maintained at 37°C, 95% air humidity, and 5% CO_2_, taken from Cell Bank of Typical Culture Preservation Committee of Chinese Academy of Sciences. The cells were treated with 100 mM ethanol or control for 24 h together with medicated serum of QGHXR, QGR, and HXR (100 mg/L). The description of specific ALI cell model methods and dose selection of medicated serum is shown in Supplementary Material. Then, the cells were harvested and collected for RNA and total protein extraction.

### Triglyceride Detection

Cell supernatant was extracted (2 × 10^4^ cells per well) after 24 h incubation and detected according to the instructions in the intracellular TG kit (A110-1, Jiancheng, Nanjing, China).

### qRT-PCR

Total RNA was extracted from both *in vivo* and *in vitro* using the Total RNA Extraction kit (19211ES60, YEASEN, Shanghai, China). The reverse transcript (cDNA) was transcribed from total RNA according to the Fast King-RT Super Mix kit (Tiangen, Beijing, China). qRT-PCR was performed with the SYBR Green PCR Master Mix (11201E03, YEASEN, Shanghai, China). Results were quantified by the 2^−ΔΔCt^ method relative to the housekeeping gene actin. Primer pairs are listed in Supplementary Table S1.

### Western Blotting

The protein samples of liver tissue and cells are carried out with phenylmethylsulfonylfluoride (PMSF) (WB0122, Weiao, Shanghai, China) containing the radioimmunoprecipitation assay (RIPA) buffer (WB0102, Weiao, Shanghai, China). Then, the protein concentration was determined by a bicinchoninic acid (BCA) protein assay kit (B48110, YEASEN, Shanghai, China). Equal protein concentrations from liver tissue or cell homogenates at 30 ug of total protein were separated by sodium dodecyl sulfate–polyacrylamide (SDS-PAGE) gel electrophoresis (8–12%) and transferred onto a 0.22-um polyvinylidene fluoride membrane. Detailed information of antibodies and dilution ratio are listed in Supplementary Table S2. Then, membranes were incubated with the secondary horseradish peroxidase-conjugated antibody (1:5,000, peroxidase-conjugated affinity goat anti-mouse IgG, HA1001, HUABIO, China) for 2 h at room temperature. Washing steps were performed between all the steps, three times each for 10 min with 1X Tris-buffered saline (TBS) (ST665, Beyotime biotechnology, Shanghai, China) containing Tween 20 buffer (ST825, Beyotime biotechnology, Shanghai, China). The bands were visualized with electrochemiluminescence (ECL) detection reagents (SB-WB012, Share bio, Shanghai, China). ImageJ software (National Institutes of Health, Bethesda, MD) was used to analyze the expression of related protein.

### Statistical Analysis

Categorical data were described using descriptive statistics (proportions and percentages). Continuous data were described using means ± SEM. A statistical analysis of data with more than two groups was evaluated by one-way analysis of variance. Data from two groups were analyzed by two-tailed Student’s t-test. *p* value <0.05 was considered as statistical significance. All data analysis was performed using SPSS version 22 (SPSS, Chicago, United States). GraphPad Prism 6.0 (GraphPad Software Inc., United States) was used to perform histograms among groups.

## Results

### The Protective Effect of QGHXR Against Alcohol-Induced Liver Injury in C57BL/6 Mice Involved Modulation of LXRα and LPCAT3

**FIGURE 1:**
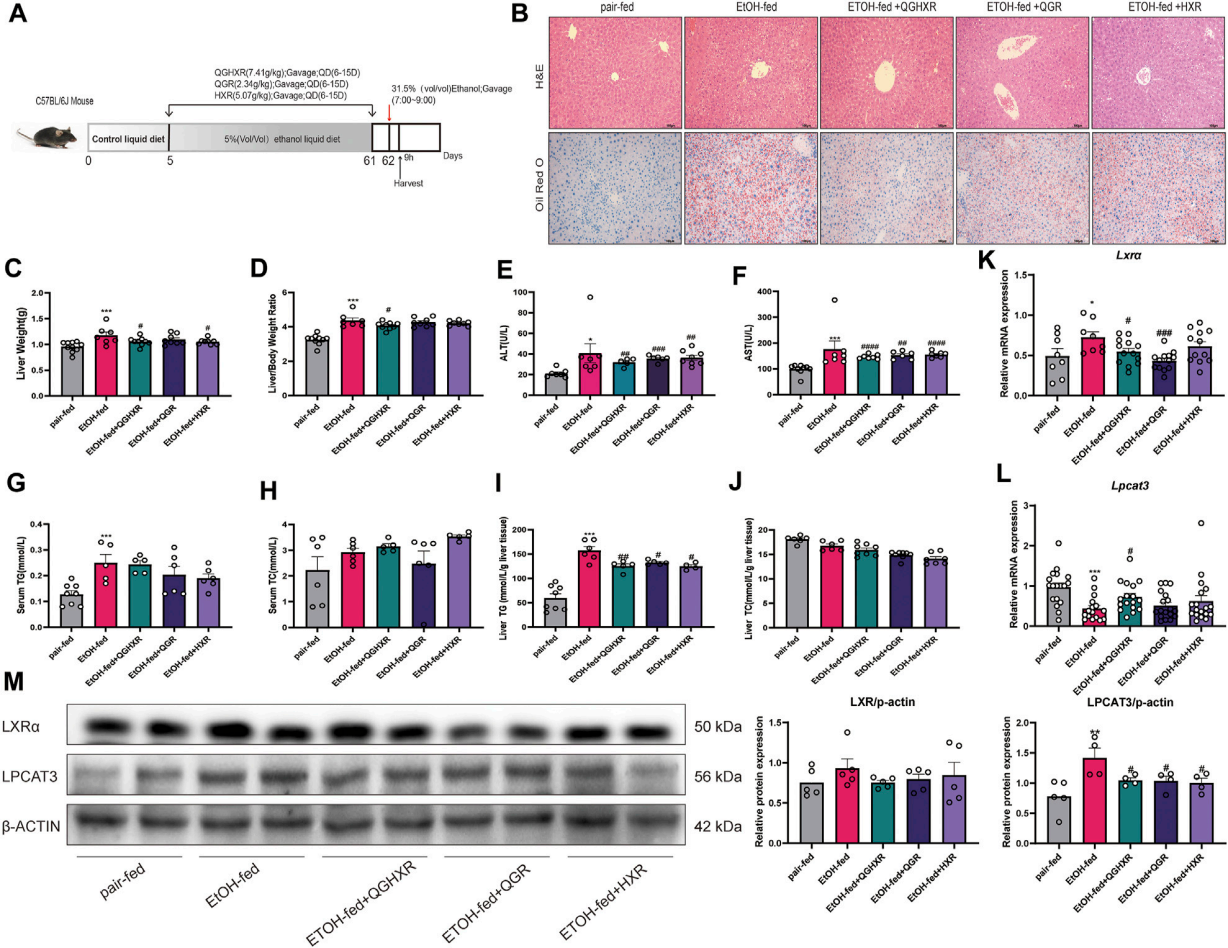
Protective effect of QGHXR against alcohol-induced liver injury in C57BL/6 mice involved modulation of LXRα and LPCAT3. **(A)** Overview of the alcohol-induced liver injury mouse model procedure. **(B)** Representative photomicrographs of liver tissue with H&E and oil red O staining (×400, 100 μm). **(C)** Liver weight (g). **(D)** Liver/body weight ratio. **(E)** Serum level of alanine transaminase (ALT) (U/L). **(F)** Serum level of aspartate transaminase (AST) (U/L). **(G)** Serum level of cytoplasmic triglyceride (TG) (mmol/L). **(H)** Serum level of total cholesterol (TC) (mmol/L). **(I)** Liver level of cytoplasmic TG (mmol/L/g liver tissue). **(J)** Liver level of TC (mmol/L/g liver tissue). **(K)** mRNA expression of *Lxr* in liver tissues. **(L)** mRNA expression of *Lpcat3* in liver tissues. **(M)** The protein expression of LXR and LPCAT3 in liver tissues. Data were presented as means ± SEM. The pair-fed group; EtOH-fed: ethanol-fed group; EtOH-fed + QGHXR: Qinggan Huoxue Recipe group with ethanol-fed; EtOH-fed + QGR: Qinggan Recipe group with ethanol-fed; EtOH-fed + HXR: Huoxue Recipe group with ethanol-fed. Quantification of mRNA and protein level expression was normalized to β-actin levels. Compared with the control group, *p < 0.05, **p < 0.01, ***p < 0.001; Compared with the EtOH-fed group, ^#^ *p* < 0.05, ^##^ *p* < 0.01, ^###^ *p* < 0.001.

First, the establishment of a stable and ideal ALI model is the basis for studying the pathogenesis of ALI. This model is given 8-weeks *ad libitum* oral feeding with the Lieber–Decarli ethanol liquid diet plus a single binge ethanol feeding (31.5%) according to the Bertola et al. (2013b) (Figure 1A). HE staining showed that compared with the pair-fed group, EtOH-fed mice showed steatosis dominated by vesicle fat, increased necrosis of hepatocytes, and inflammatory cell infiltration in the liver sinusoids, and each medication group had different degrees of improvement. The oil red O staining showed that a large number of lipid droplets were formed in the liver tissue of mice in the EtOH-fed group with hepatocyte swelling. In addition, the significantly upregulated cytochromes P450 2E1 (CYP2E1) protein expression from immunohistochemical staining and increased Il-6 and monocyte chemoattractant protein-1 (*Mcp-1*) mRNA expression from qRT-PCR in the EtOH-fed group than those in the pair-fed group indicated that alcohol caused severe liver damage (Supplementary Figures S1A,B). However, although the expression of CYP2E1 in the medication group was lower than that in the EtOH-fed group, there was no statistical difference (Supplementary Figure S1C). The mRNA expression of *Il-6* and *Mcp-1* was decreased in the medication group compared to the EtOH-fed group (Supplementary Figure S1B). Compared with the EtOH-fed group, inflammatory cell infiltration was not significantly decreased by QGHXR through HE staining, but fat deposition was significantly improved after treatment with QGHXR through oil red O staining (Figure 1B). Our results first confirmed that QGHXR improved liver injury in EtOH-fed mice including decreased liver weight (*p* < 0.05, Figure 1C), liver/body weight ratio (*p* < 0.05, Figure 1D), serum ALT (*p* < 0.01, Figure 1E), AST (*p* < 0.01, Figure 1F), and liver TG (*p* < 0.01, Figure 1I), while QGR improved liver injury by decreasing serum ALT (*p* < 0.001), AST (*p* < 0.01), liver TG (*p*<0.05), and HXR by decreasing serum ALT (*p* < 0.01), serum AST (*p* < 0.001), and liver TG (*p* < 0.05). Both serum and liver TC showed no obvious downward trend after 8-week intervention by QGHXR (Figures 1H,J). From the mRNA level and protein level, it was shown with significant changes in LXRα and LPCAT3 at the transcriptional level, suggesting that ethanol stimulation broke the steady-state of ER and the recovery of related factors of QGHXR may be the potential mechanism of its improvement of ALI. Compared with the pair-fed group, mRNA expression of *Lxrα* was significantly increased in the EtOH-fed group (*p* < 0.05). QGHXR (*p* < 0.05) and QGR (*p* < 0.01) can significantly inhibit mRNA expression of *Lxrα*, except HXR (Figure 1K). *Lpcat3* mRNA expression decreased significantly in the EtOH-fed group (*p* < 0.001) compared with the control group. Only QGHXR significantly promoted its expression (*p* < 0.05) (Figure 1L). There was no significant difference in the protein level of LXRα among the groups either from WB (Figure 1M) or IHC (Supplementary Figures S2A,C). The protein expression of LPCAT3 was drastically increased in the EtOH-fed group (*p* < 0.01), and each medication group could significantly inhibit its protein expression (*p* < 0.05) (Figure 1M). Furthermore, QGHXR, QGR, and HXR improved ALI by regulating the mRNA expression of fatty acid oxidation related factors such as peroxisome proliferator-activated receptor-α (PPAR-α) and carnitine palmitoyltransferase 1 (CPT1) (*p* < 0.05); less relationship with sterol regulatory element binding transcription factor 1(SREBP1) was observed (Supplementary Figure S2B).

### Protective Effect of QGHXR Against Alcohol-Induced Liver Injury in C57BL/6 Mice Involved Modulation of Factors Related to ERS

**FIGURE 2:**
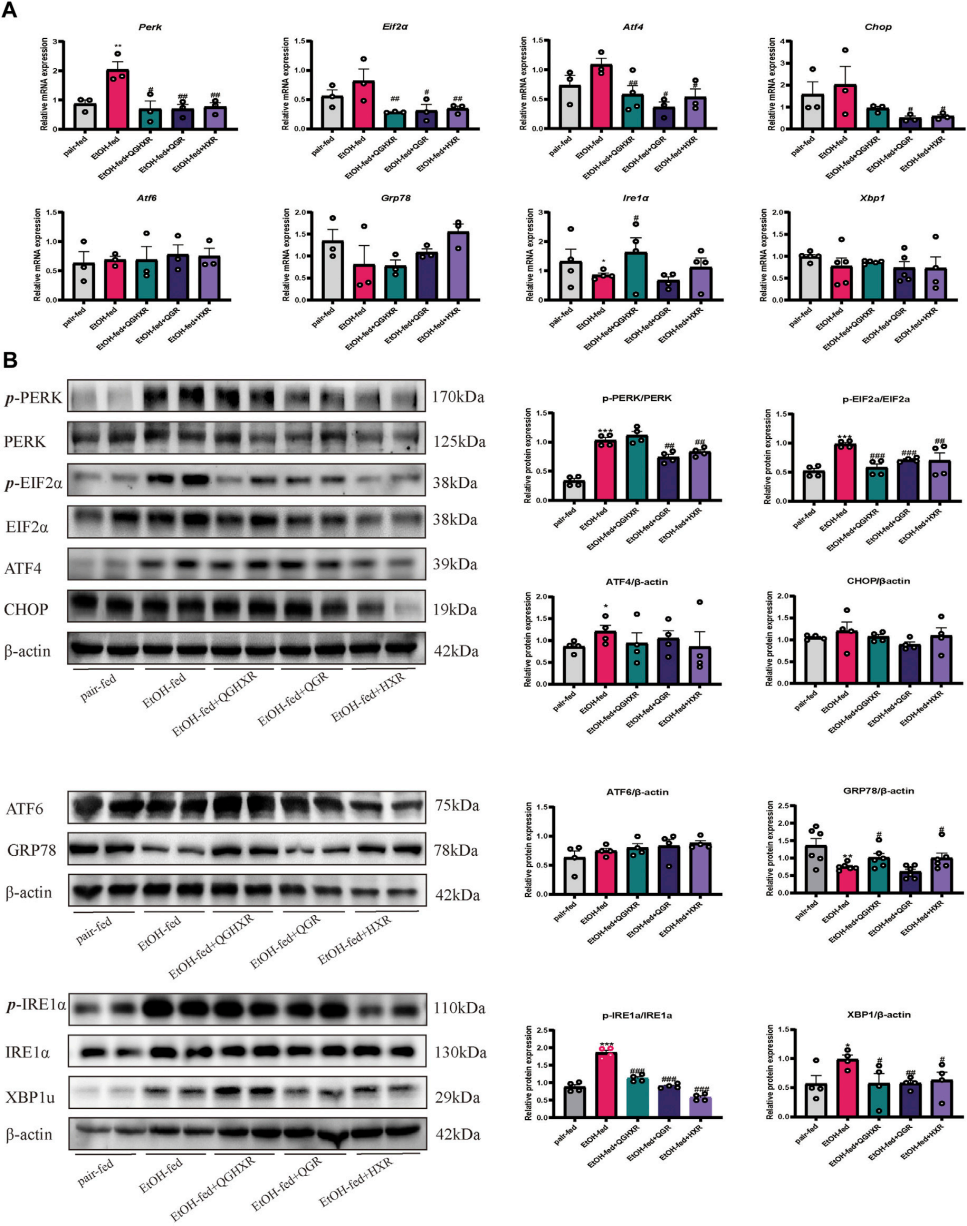
Protective effect of QGHXR against alcohol-induced liver injury in C57BL/6 mice involved modulation of factors related to ERS. **(A)** mRNA expression in liver samples; **(B)** protein expression in liver samples. The pair-fed group; EtOH-fed: ethanol-fed group; EtOH-fed + QGHXR: Qinggan Huoxue Recipe group with ethanol-fed; EtOH-fed + QGR: Qinggan Recipe group with ethanol-fed; EtOH-fed + HXR: Huoxue Recipe group with ethanol-fed. For **(A,B)**, quantification of mRNA and protein level expression was normalized to β-actin levels, except protein phosphorylation level expression of PERK, eIF2α, and IRE1α was normalized to their prototypes. Data were presented as means ± SEM. Compared with the pair-fed group, *p < 0.05, **p < 0.01, ***p < 0.001; Compared with the EtOH-fed group, ^#^ *p* < 0.05, ^##^ *p* < 0.01, ^###^ *p* < 0.001.

Second, in order to further verify whether the mechanism of QGHXR on improving ALI was related to ERS, we detected the expression of mRNA and protein of ERS-related factors. Compared with the pair-fed group, mRNA expression of *Ire1α* were significantly decreased in the EtOH-fed group (*p* < 0.05), while mRNA expression of *Perk* was significantly increased (*p* < 0.01) (Figure 2A). Compared with the pair-fed group, protein expression of PERK (*p* < 0.001), eIF2α (*p* < 0.001), ATF4 (*p* < 0.05), IRE1α (*p* < 0.001), and XBP1u (*p* < 0.05) were significantly increased in the EtOH-fed group, while GRP78 (*p* < 0.01) protein expression was significantly decreased (Figure 2B). QGHXR mainly inhibited mRNA expression of *Perk* (*p* < 0.05), *Eif2α* (*p* < 0.01), and *Atf4* (*p* < 0.01) and activated the mRNA expression of *Ire1α* (*p* < 0.05) (Figure 2A), while it inhibited protein expression of eIF2α (*p* < 0.001), IRE1α (*p* < 0.001), and XBP1u (*p* < 0.05) and activates protein expression of GRP78 (*p* < 0.05) to improve ALI (Figure 2B). QGR was more inclined to improve chronic ALI by inhibiting mRNA expression of *Perk* (*p* < 0.01), *Eif2α* (*p* < 0.05), and *Atf4* (*p* < 0.05) (Figure 2A) and inhibiting protein expression of PERK (*p* < 0.01), eIF2α (*p* < 0.001), IRE1α (*p* < 0.001), and XBP1u (*p* < 0.01) (Figure 2B). HXR was more inclined to improve ALI by inhibiting mRNA expression of *Perk* (*p* < 0.01), *Eif2α* (*p* < 0.01), *Atf4* (*p* < 0.05), and *Chop* (*p* < 0.05) (Figure 2A), inhibiting protein expression of PERK (*p* < 0.01), eIF2α (*p* < 0.01), IRE1α (*p* < 0.001), XBP1u (*p* < 0.05), and activating protein expression of GRP78 (*p* < 0.05) (Figure 2B). The detailed modulation of QGHXR against ALI in C57BL/6 mice is summarized in Table 1.

**TABLE 1:** Protective effect of QGHXR against alcohol-induced liver injury in C57BL/6 mice through the LXRα-LPCAT3-ERS pathway.

		ALI in C57BL/6 mice			
		M vs. C	QGHXR vs. M	QGR vs. M	HXR vs. M
PCR	*Lxrα*	↑	↓	↓	—
	*Lpcat3*	↓	↑	—	—
	*Perk*	↑	↓	↓	↓
	*Eif2α*	—	↓	↓	↓
	*Atf4*	—	↓	↓	↓
	*Chop*	—	—	↓	↓
	*Atf6*	—	—	—	—
	*Grp78*	—	—	—	—
	*Ire1α*	↓	↑	—	—
	*Xbp1*	—	—	—	—
WB	LXR/β-actin	—	—	—	—
	LPCAT3/β-actin	↑	↓	↓	↓
	*p*-PERK/PERK	↑	—	↓	↓
	*p*-EIF2α/EIF2α	↑	↓	↓	↓
	ATF4/β-actin	↑	—	—	—
	CHOP/β-actin	—	—	—	—
	ATF6/β-actin	—	—	—	—
	GRP78/β-actin	↓	↑	—	↑
	*p*-IRE1α/IRE1α	↑	↓	↓	↓
	XBP1u/β-actin	↑	↓	↓	↓

C, pair-fed group; M, EtOH-fed group; QGHXR, EtOH-fed + Qinggan Huoxue Recipe group; QGR, EtOH-fed + Qinggan Recipe group; HXR, EtOH-fed + Huoxue Recipe group.

### QGHXR Regulates Lipid Accumulation and Inflammation in Ethanol-Induced Liver Injury Through Modulating of LXRα and LPCAT3

**FIGURE 3:**
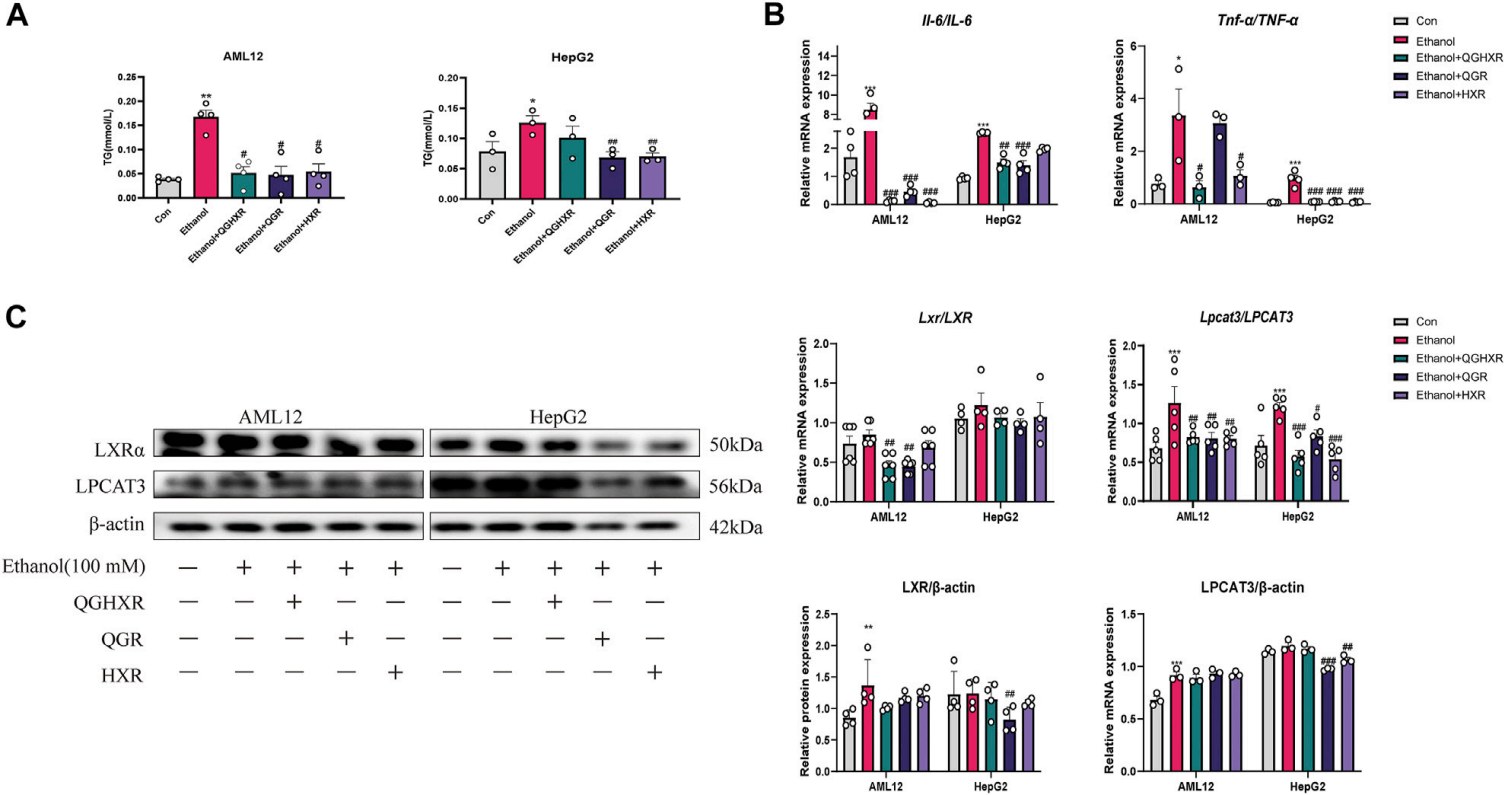
Protective effect of QGHXR against alcohol-induced liver injury in AML12 and HepG2 cell involved modulation of LXRα and LPCAT3. **(A)** Level of TG (mmol/L) in AML12 and HepG2 cell. **(B)** mRNA expression of *Il-6* and *Tnf-α* in AML12 cell and HepG2 cell. **(C)** mRNA and protein expression of LXR and LPCAT3 in AML12 and HepG2 cell. Con: normal control group, ethanol: ethanol group (100 mM), ethanol + QGHXR: Qinggan Huoxue Recipe group induced by ethanol; ethanol + QGR: Qinggan Recipe group induced by ethanol; ethanol + HXR: Huoxue Recipe group induced by ethanol. Quantification of mRNA and protein level expression was normalized to β-actin levels. Data were presented as means ± SEM. Compared with the normal control group, *p < 0.05, **p < 0.01, ***p < 0.001; Compared with the ethanol group, ^#^ *p* < 0.05, ^##^ *p* < 0.01, ^###^ *p* < 0.001.

In order to further prove the efficacy of QGHXR on the ALI model, we further used the *in vitro* model. First, a liver injury cell model (100 mM ethanol) *in vitro* was established and best dosage of QGHXR-, QGR-, and HXR-medicated serum (100 mg/L) in cells were selected (Supplementary Figure S3). Compared with the control group, TG content in AML12 and HepG2 cells in the ethanol group was significantly increased (*p* < 0.01 and *p* < 0.05). The TG content of AML12 cells was significantly decreased after QGHXR, QGR, and HXR treatment (*p* < 0.05), and TG in HepG2 cell was significantly decreased after QGR and HXR treatment (*p* < 0.01) (Figure 3A). The mRNA levels of IL-6 and TNF-α were substantially upregulated in both HepG2 and AML12 cells induced by ethanol (*p* < 0.05, *p* < 0.001). After QGHXR, QGR, and HXR intervention, the mRNA transcription of IL-6 and TNF-α were significantly downregulated in both cells (*p* < 0.05, *p* < 0.001) (Figure 3B). In AML12 cells, *Lxrα* mRNA expression was significantly increased in the ethanol group compared with control group, while QGHXR (*p* < 0.01) and QGR (*p* < 0.01) can significantly inhibit its expression, but HXR has no obvious effect. In HepG2 cells, compared with the control group, mRNA expression of *LXRα* was substantially increased in the ethanol group, and there was no significant increase in each medication group (Figure 3C). Western blot results showed that in AML12 cells, the protein expression of LXRα in the ethanol group was significantly higher than that of the control group (*p* < 0.01), and there was no significant difference between other two groups. In HepG2 cells, compared with the control group, the protein expression of LXRα in the ethanol group was significantly increased, and QGR (*p* < 0.05) can significantly inhibit its expression. In AML12 cells, the protein expression of LPCAT3 in the ethanol group was significantly higher than that in the control group (*p* < 0.001), and there was no significant difference between other two groups. In HepG2 cells, QGR (*p* < 0.001) and HXR (*p* < 0.01) can significantly inhibit its protein expression (Figure 3C).

### QGHXR Regulates Liver Injury Cells Induced by Ethanol Through Modulating of Factors Related to ERS

**FIGURE 4:**
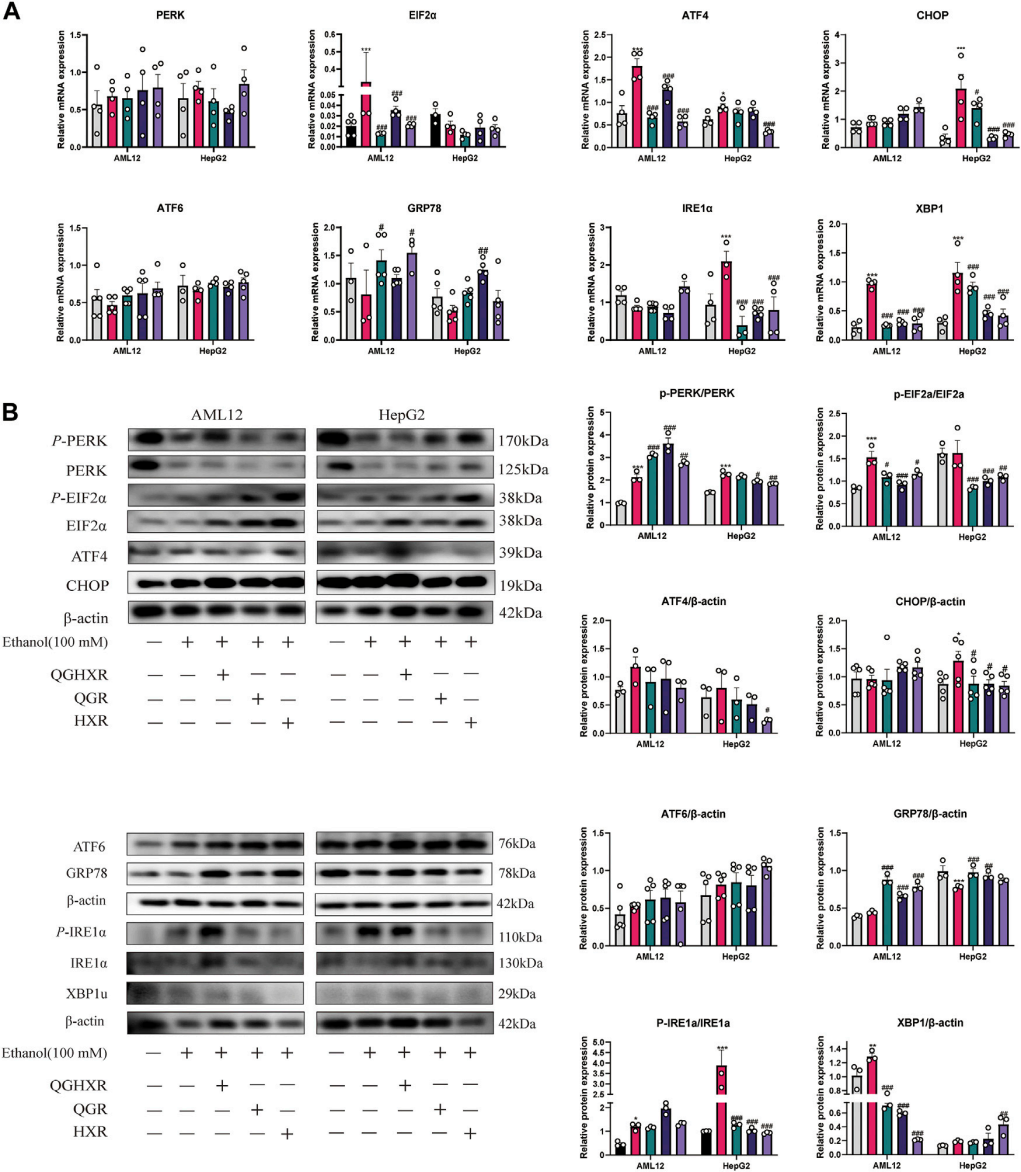
Protective effect of QGHXR against alcohol-induced liver injury in AML12 and HepG2 cell involved modulation of factors related to ERS. **(A)** mRNA expression in both cell samples; **(B)** protein expression in both cell samples. Con: normal control group, ethanol: ethanol group (100 mM), ethanol + QGHXR: Qinggan Huoxue Recipe group induced by ethanol; ethanol + QGR: Qinggan Recipe group induced by ethanol; ethanol + HXR: Huoxue Recipe group induced by ethanol. For **(A,B)**, quantification of mRNA and protein level expression was normalized to β-actin levels, except protein phosphorylation level expression of PERK, eIF2α, and IRE1α was normalized to their prototypes. Data were presented as means ± SEM. Compared with the normal control group, *p < 0.05, **p < 0.01, ***p < 0.001; Compared with the ethanol group, ^#^ *p* < 0.05, ^##^ *p* < 0.01, ^###^ *p* < 0.001.

In AML12 cells, the mRNA expression of *Eif2α* (*p* < 0.001), *Atf4* (*p* < 0.001), and *Xbp1* (*p* < 0.001) in the ethanol group was increased significantly, and QGHXR, QGR, and HXR could significantly inhibit its expression (*p* < 0.001). However, this trend of *Eif2α* was not obvious and only HXR can significantly inhibit *ATF4* (*p* < 0.001) mRNA expression in HepG2 cell (Figure 4A). In HepG2 cells, compared with the control group, the mRNA expression of *CHOP* in the ethanol group was significantly increased (*p* < 0.001). QGHXR (*p* < 0.05), QGR (*p* < 0.01), and HXR (*p* < 0.01) were all able to inhibit its expression (*p* < 0.001). There was no significant difference in *Atf6* and *ATF6* mRNA levels among three groups of in both cells. In AML12 cells, compared with the ethanol group, QGHXR (*p* < 0.05) and HXR (*p* < 0.05) can significantly promote the mRNA expression of *Grp78* mRNA, while QGR can significantly promote it compared to the ethanol group in HepG2 cells (*p* < 0.01). In HepG2 cells, the mRNA expressions of *IRE1α* in the ethanol group were significantly higher than those in the control group, and QGHXR, QGR, and HXR could significantly inhibit their expression (*p* < 0.001) (Figure 4A).

In terms of protein expression, in AML12 cells, the phosphorylation level of PERK and eIF2α in the ethanol group was significantly higher than that in the control group (*p* < 0.001), and the phosphorylation level of PERK in all three groups was significantly promoted, while QGHXR (*p* < 0.05), QGR (*p* < 0.001), and HXR (*p* < 0.05) can inhibit the phosphorylation level of eIF2α. In HepG2 cells, the phosphorylation level of PERK (*p* < 0.001) and the protein of CHOP (*p* < 0.05) in the ethanol group were significantly higher than that in the control group. Compared with the ethanol group, QGR (*p* < 0.01) and HXR (*p* < 0.001) can significantly inhibit the phosphorylation level of PERK, while QGHXR (*p* < 0.001), QGR (*p* < 0.001), and HXR (*p* < 0.01) can inhibit the phosphorylation level of eIF2α and the protein of CHOP (*p* < 0.05). In HepG2 cells, compared with the ethanol group, HXR could significantly inhibit the protein expression of ATF4 (*p* < 0.05). There was no significant difference in the protein level of ATF6 in both cells. In AML12 cells, compared with the ethanol group, QGHXR, QGR, and HXR could significantly promote the expression of GRP78 (*p* < 0.001). In HepG2 cells, compared with the control group, the protein expression of GRP78 in the ethanol group was significantly decreased (*p* < 0.001), and QGHXR (*p* < 0.001) and QGR (*p* < 0.01) could significantly promote its expression. In addition, the phosphorylation level of IRE1α in the ethanol group was higher than that in the control group both in AML12 cell (*p* < 0.05) and HepG2 cell (*p* < 0.001), and the expression of phosphorylation was significantly inhibited in all three treatment groups (*p* < 0.001) in HepG2 cell. In AML12 cells, the protein expression of XBP1u in the ethanol group was significantly higher than that in the control group (*p* < 0.01), and all three treatment groups could significantly inhibit its expression (*p* < 0.01). The protein expression of XBP1u in HXR was significantly promoted compared with that in the ethanol group (*p* < 0.01) (Figure 4B). The detailed modulation of QGHXR against ALI in AML12 and HepG2 is summarized in Table 2.

**TABLE 2:** Protective effect of QGHXR against alcohol-induced liver injury in AML12 and HepG2 cell through the LXRα-LPCAT3-ERS pathway.

		AML12				HepG2			
		M vs. C	QGHXR vs. M	QGR vs. M	HXR vs. M	M vs. C	QGHXR vs. M	QGR vs. M	HXR vs. M
PCR	*Lxrα/LXRα*	—	↓	↓	—	—	—	—	—
	*Lpcat3/LPCAT3*	↑	↓	↓	↓	↑	↓	↓	↓
	*Perk/PERK*	—	—	—	—	—	—	—	—
	*Eif2α/EIF2α*	↑	↓	↓	↓	—	—	—	—
	*Atif4/ATF4*	↑	↓	↓	↓	↑	—	—	↓
	*Chop/CHOP*	—	—	—	—	↑	↓	↓	↓
	*Atf6/ATF6*	—	—	—	—	—	—	—	—
	*Grp78/GRP78*	—	↑	—	↑	—	—	↑	—
	*Ire1α/IRE1α*	—	—	—	—	↑	↓	↓	↓
	*Xbp1/XBP1*	↑	↓	↓	↓	↑	↓	↓	↓
WB	LXR/β-actin	↑	—	—	—	—	—	↓	—
	LPCAT3/β-ctin	↑	—	—	—	—	—	↓	↓
	*p*-PERK/PERK	↑	↑	↑	↑	↑	—	↓	↓
	*p*-EIF2α/EIF2α	↑	↓	↓	↓	—	↓	↓	↓
	ATF4/β-actin	—	—	—	—	—	—	—	↓
	CHOP/β-actin	—	—	—	—	↑	↓	↓	↓
	ATF6/β-actin	—	—	—	—	—	—	—	—
	GRP78/β-actin	—	↑	↑	↑	↓	↑	↑	↑
	*p*-IRE1/IRE1α	↑	—	—	—	↑	↓	↓	↓
	XBP1u/β-actin	↑	↓	↓	↓	—	—	—	↑

C, normal control group; M, ethanol group; QGHXR, ethanol + Qinggan Huoxue Recipe group; QGR, ethanol + Qinggan Recipe group; HXR, ethanol + Huoxue Recipe group.

## Discussion

Liver is the most common target organ of alcohol toxicity (Arteel, 2003). It was shown that the prevalence of fatty liver caused by alcohol was as high as 4.7% in 2016 (Wong et al., 2019). Among them, more than 80% excessive drinking will produce fatty liver, of which 10–35% can develop into alcoholic hepatitis (Campollo et al., 2001). Therefore, it is important to timely prevent the development of ALI.

LXR is a nuclear cholesterol-sensing transcription factor that plays an important role in regulating fatty acid, cholesterol, and glucose metabolism (Gavini et al., 2018). Our previous study also found that downregulation of LXRα could improve liver lipid accumulation (Xiang et al., 2021). The similar mRNA results have been shown in our experiments, especially in animals, both *in vivo* and in AML12 cell. However there were no significant differences of the protein expression of LXRα among groups either WB or IHC detection.

LPCAT3 is a key molecule regulating intracellular PLs regulated by LXR, which regulates the formation of ERS and inflammation (Rong et al., 2013). As a direct target gene of LXR, LPCAT3 preferentially synthesized PC containing unsaturated fatty acids, such as arachidonic acid (20:4) and linoleic acid (18:2) at sn-2 position (Lands, 2000) of acylation of unsaturated fatty acids (Li et al., 2012). Some scholars believe that LPCAT3 can incorporate polyunsaturated fatty acids into phospholipids to promote the processing and lipogenesis of SREBP-1c (Liu et al., 2003). On the contrary, hepatocyte-deficient lpcat3 mice can reduce the level of polyunsaturated phospholipids in ER membrane, reduce the level of SREBP-1c in nucleus, and slow down the lipogenic response when treated with LXR agonists (Walker et al., 2011; Rong et al., 2015). The role of LPCAT3 in maintaining the homeostasis of ER membrane under lipid stress in different physiological and pathological environments needs to be further studied. However, the key role of LPCAT3 in maintaining lipid homeostasis, ERS, and inflammation is obvious. Our experiment support that increasing the protein level of LPCAT3 in liver tissue of ALI mice may be more related to the promotion of fat production by LPCAT3, and the results were also confirmed *in vitro* experiments.

**FIGURE 5:**
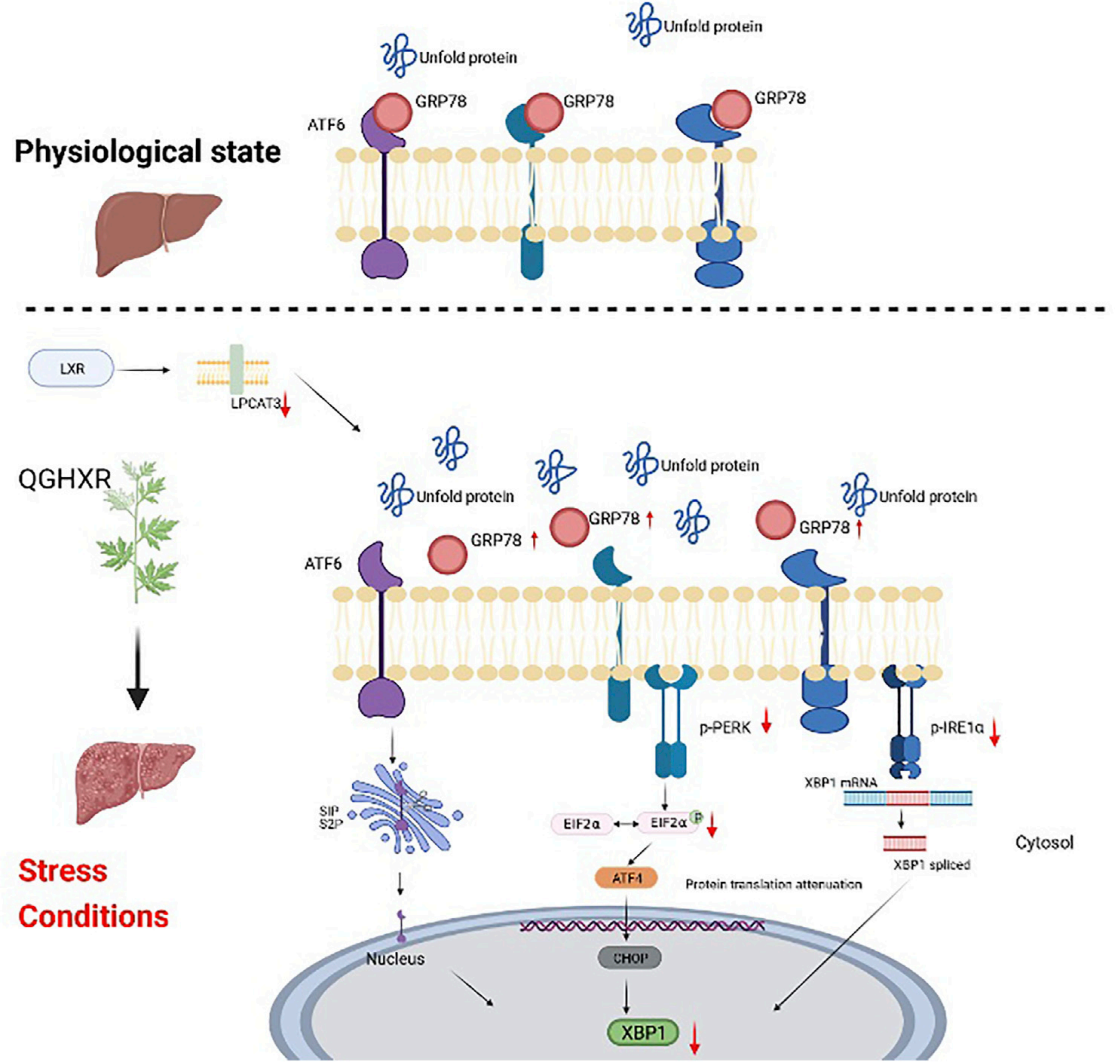
Schematic diagram of the mechanism of QGHXR regulating the LXRα-LPCAT3 signaling pathway. It was created in the website www.BioRender.com. QGHXR: Qinggan Huoxue Recipe group; LPCAT3: lysophosphatidylcholine acyltransferase 3; LXR α: liver X receptor alpha; PERK: protein kinase R-like ER kinase; eIF2α: eukaryotic translation initiation factor 2α; ATF6: activating transcription factor 6; IRE1: inositol-requiring enzyme 1; XBP1: X-box-binding protein 1; ATF4: activating transcription factor 4; CHOP: C/EBP homologous protein.

ER plays an important role in the stability of the intracellular environment and the synthesis, modification, secretion, and folding of proteins (Rutkowski and Kaufman, 2004). When excessive unfolded protein accumulates in the lumen and Ca+ is disturbed, a protective response called unfolded protein response (UPR) is triggered to respond to ERS. However, when too much UPR was coped with ERS, ERS can finally lead to a variety of pathological consequences, including liver fat accumulation, inflammation, and apoptosis (Ji and Kaplowitz, 2006) and activate PERK/elF2α, ATF6, and IRE1/XBP-1 signaling pathways along with dissociation of GRP78 from ER (Ye et al., 2000; Liu et al., 2003). The self-phosphorylation of PERK activates and phosphorylates eIF2α, thus protein synthesis is inhibited to reduce UPR overload and ERS (Hetz and Glimcher, 2009) along with the upregulated UPR target gene ATF4 and the downstream target gene CHOP (Malhi and Kaufman, 2011). After self-phosphorylation of IRE1α, the RNA enzyme activity is activated and XBP1 gene splicing is initiated, resulting in an active transcription factor XBP1s and a non-splicing body XBP1u. XBP1s controls protein folding, redox reaction, and UPR upregulation and has a great relationship with cell differentiation and DNA damage (Hetz and Glimcher, 2009). XBP1u is a precursor of XBP1s, and its deletion can significantly induce cell cycle g0-G1 phase arrest and inhibit cell proliferation, thus significantly reducing tumor formation of tumor cells (Huang et al., 2017). The accumulation of unfolded proteins also leads to the transport of ATF6 to the Golgi complex, where it is cut by site-1 protein (S1P) and site-2 protein (S2P). Free cytoplasmic domains as active transcription factors (Ye et al., 2000) are produced to activate the transcription of XBP1. Combined with the results of *in vivo* experiments, it is suggested that QGHXR can improve the imbalance of the LXRα-LPCAT3 pathway and prevent further development of ERS, which is mainly related to PERK-eIF2α and IRE1α, and a relatively small relationship with the ATF6 pathway (Figure 5).

The decreased expression of GRP78 also leads to the lack of sufficient GRP78 chaperone proteins in the downstream ERS to bind with them to form a stable and firm ER homeostatic state, resulting in more severe ERS and leading to severe liver injury (Ji et al., 2011). We found that the expression of GRP78 can be significantly increased through QGHXR, and the mechanism may be associated with the protective mechanism of ER by improving its expression (Maiers and Malhi, 2019). The detailed mechanism how GRP78 is expressed in the occurrence and development of ERS may be the focus of future research.

At the cellular level, PCR and western blot results showed that the mRNA and protein levels of LXRα and Lpcat3 were significantly increased in the ethanol-induced ALI model cells compared with the control group, suggesting that the stimulation of alcohol can result in imbalance of the LXRα-LPCAT3 pathway. QGHXR can significantly inhibit the expression of LPCAT3 mRNA to alleviate the ethanol-induced ALI. The mRNA and protein expression of ERS-related molecules eIF2α and PERK were significantly increased in ALI model cells compared with the control group. In the ALI model of AML12 cells, the mRNA and protein expressions of eIF2α and XBP1 were significantly increased, and the expression of PERK protein and ATF4 mRNA were significantly increased. In the ALI model of HepG2 cells, the expression of CHOP and IRE1α protein and mRNA was mainly increased, and the expression of PERK protein and ATF4 and XBP1 mRNA was increased. After the treatment with drug-containing serum of QGHXR, the mRNA and protein expressions of ERS-related factors tended to return to normal levels, and the pathways regulated by QGHXR were also different in different cell lines. QGHXR can effectively alleviate ALI by inhibiting the further development of ERS.

### Limitation of the Study

There are still several limitations in the present study. First, the dynamic model *in vivo* animal experiment with different alcohol concentration and intervention time can be used to select a more appropriate and accurate animal model. Second, whole body-knockout or liver-specific knockout mice by CRISPR/CAS9 can used to further verify the key role of LPCAT3 and the exact mechanism of how QGHXR regulates ERS through the LXRα-LPCAT3 pathway to improve ALI. Finally, it should be noted that the animal model used in this research could not fully reflect the react human diseases. Therefore, our further experiments will further focus on the improvement effect and mechanism of the QGHXR on ALI patients.

## Conclusion

In conclusion, this study confirmed that QGHXR can improve alcohol liver injury *in vivo* and alleviate the damage of ethanol on liver cells *in vitro*, mainly by regulating LXRα and LPCAT3 expression levels to improve ERS, and then alleviate liver steatosis inflammation and liver damage caused by alcohol.

## Data Availability

The original contributions presented in the study are included in the article/Supplementary Material, further inquiries can be directed to the corresponding authors.

## Abbreviations

AC, alcoholic cirrhosis; AFL, alcoholic fatty liver; AH, alcohol hepatitis; AHF, alcoholic hepatic fibrosis; ALI, alcohol liver injury; ALT, alanine transaminase; AST, aspartate transaminase; ATF4, activating transcription factor 4; ATF6, activating transcription factor 6; CHOP, C/EBP homologous protein 10; eIF2α, eukaryotic translation factor 2; ERS, endoplasmic reticulum stress; GRP78, glucose-regulated protein 78; HXR, Huoxue Recipe; IRE1α, inositol-requiring enzyme 1α; LPCAT3, lysophosphatidylcholine acyltransferase 3; LXRα, liver X receptor alpha; MAI, mild alcoholic injury; PERK, PKR-like endoplasmic reticulum kinase; QGHXR, Qinggan Huoxue Recipe; QGR, Qinggan Recipe; RT-qPCR, real-time-quantitative polymerase chain reaction; TG, triglyceride; TC, total cholesterol; and XBP1, X-box-binding protein 1.
