# Supplementary material for: Qinggan Huoxue Recipe Alleviates Alcoholic Liver Injury by Suppressing Endoplasmic Reticulum Stress Through LXR-LPCAT3
Source: Front Pharmacol. 2022 Mar 31;13:824185. doi: 10.3389/fphar.2022.824185 (PMC9009225; doi:10.3389/fphar.2022.824185)
Supplement: Supplementary file 1 [file DataSheet1.docx]

# Supplementary Material

**Qinggan Huoxue Recipe alleviates chronic alcoholic liver injury by suppressing endoplasmic reticulum stress through LXR-LPCAT3**

Yifei Lu^1,3^, Mingmei Shao^1,4^, Hongjiao Xiang^1,2^, Junmin Wang^1,2^, Guang Ji^2^*and Tao Wu^1,2^*

1 Institute of Interdisciplinary Integrative Medicine Research, Shanghai University of Traditional Chinese Medicine, Shanghai 201203, China

2 Institute of Digestive Disease, Longhua Hospital, Shanghai University of Traditional Chinese Medicine, Shanghai 200032, China.

3 Yueyang Hospital of Integrated Traditional Chinese and Western Medicine, Shanghai University of Traditional Chinese Medicine, Shanghai 200083, China

4 Teaching Department, Baoshan District Hospital of Intergrated Traditional Chinese and Western Medicine,181 You-yi Road, Shanghai 201900, China.

*co-corresponding author.

**Correspondence to:** Dr. Tao Wu, PhD. Institute of Interdisciplinary Integrative Medicine Research, Shanghai University of Traditional Chinese Medicine, Cailun Road 1200, Shanghai 201203, China. Email: tw827@shutcm.edu.cn or wutao001827@163.com; Prof. Guang Ji, PhD. Institute of Digestive Disease, Longhua Hospital, Shanghai University of Traditional Chinese Medicine, South Wanping Road 725, Shanghai 200032, China. Email: jg@shutcm.edu.cn.

**Running Title:** QGHXR alleviates alcoholic liver injury

**List**

**Supplementary Methods**

1. Preparation of freeze-dried powder containing medicated serum

1.1 Animals and treatments

1.2 Collection and production of medicated serum

2. The optimal concentration of ethanol stimulation in AML12 and HepG2 cell injury models

3. Safe range of QGHXR, QGR, HXR on cells

4. Immunohistochemical staining

**Supplementary Figure 1.** Representative immunohistochemical staining pictures of CYP2E1 expression and mRNA expression of inflammatory factors in liver tissue among groups.

**Supplementary Figure 2.** Representative immunohistochemical staining pictures of LXR expression and the mRNA expression of fatty acid oxidation factors in liver tissue among groups.

**Supplementary Figure 3.** Establishment of a liver injury cell model in vitro, the best simulation condition of ethanol and the best intervention condition of QGHXR, QGR, HXR medicated serum in cells.

**Supplementary Table 1.** Primer sequences of the genes used for the qRT-PCR.

**Supplementary Table 2.** Antibodies for Western blot.

**Supplementary Methods**

**1. Preparation of freeze-dried powder containing medicated serum**

**1.1 Animals and treatments**

Specific-pathogen-free six-week-old male Wistar rats were provided by Beijing Vital River Laboratory Animal Technology Co., Ltd. and kept in the Experimental Animal Center of ShanghaiUniversity of Traditional Chinese Medicine.The experiments were conducted under the Guidelines for Animal Experiment of Shanghai University of Traditional Chinese Medicine and the protocol was approved by the institutional Animal Ethics Committee (Approval number: PZSHUTCM191213002). Rats breeding environment are indoor standard clean, standard temperature 22±2 ℃, humidity 55%~65%, regular autoclave sterilization in ventilated cage, regular replacement of bedding. After adaptive feeding, ratswere divided into four groups(n=6 per group): (1) normal control group (C); (2)QingganHuoxueRecipe group(QGHXR) (5.13 g/kg/d); (3) Qinggan Recipe group (QGR) (1.62 g/kg/d); (4)Huoxue Recipe group (HXR)(3.51 g/kg/d). All rats were fasted overnight 3 day later, and then gavage again in the morning the next day, and the material was taken 2 hours later.

**1.2 Collection and production of medicated serum**

After anesthetized by intraperitoneal injection of 0.5 ml/100 g of 7% chloral hydrate, the rats blood was taken from the abdominal aorta, placed at 4°C for 3 hours, centrifuged at 3000 rpm for 20 minutes, and the supernatant was taken and placed in a 56°C water bath for 30 minutes to inactivate. After inactivation, the ultra-clean stage is passed through a 0.22 μm filter membrane, frozen at -80 ℃ to make solid ice cubes, and serum freeze-dried powder were made by freeze-drying machine at -70 ℃ (Freeze dryer, SCIENTZ-12N/A-80), then stored in the refrigerator at -20 ℃. The specific drug-containing serum freeze-dried powder of each group is as follows:

| **Group** | **n** | **serum（ml）** | **powder（g）** |
| --- | --- | --- | --- |
| C | 6 | 17 | 1.1964 |
| QGHXR | 6 | 24 | 1.1612 |
| QGR | 6 | 22 | 1.3821 |
| HXR | 6 | 23 | 0.7789 |

**2. The optimal concentration of ethanol stimulation in AML12 and HepG2 cell injury models**

In order to determine the best modeling conditions for HepG2 and AML12 cells by ethanol concentration, the experiment was performed when the cells were in good condition and grew to about 80%. The ethanol was filtered through a 0.22 μm membrane and added to culture medium without serum. The final concentrations were 0, 50, 100, 150, 200, 300, and 500 mM, respectively. After 24 hours of observation, cell counting kit-8 (CCK8) (40203, YEASEN, Shanghai,China) measures the cell survival rate. Cell survival rate= (intervention group D-blank group D)/ (control group D-blank group D) ×100%. The cell growth curve was fitted by Graphpad Prism 8.3.0 and the half maximal inhibitory concentration (Half maximal inhibitory concentration, IC50). The experiment was repeated three times.

**3. Safe range of QGHXR**, QGR, HXR **on cells**

Firstly, six concentrations of 5, 10, 50, 100, 500, 1000 mg/L in serum of QGHXR, QGR, and HXR were established with each with 6 replicate holes. After 24 hours of observation, CCK8(40203, YEASEN, Shanghai, China) measures the cell survival rate. After in a 37°C incubator for 3 h, the optical density was measured at 450 nm wavelength with a microplate reader (Infinite M200PRO, TECAN, Switzerland). The experiment was repeated three times.

### 4. Immunohistochemical staining (IHC)

Paraffin sections were deparaffinized with xylene and dehydrated with gradient ethanol. The sections were immersed in 10 mM sodium citrate buffer (pH 6.0) and heated in microwave 10min for antigen retrieval. After cool it to room temperature, and then repeat high fire for 12 minutes. After cooling to room temperature, those sections were blocked with 5%BSA and incubated with prediluted LXR or CYP2E1 primary antibodies, followed by incubating with appropriate secondary antibodies. The bound antibodies were observed with the Lab Vision ™ DAB Plus (Thermo Fisher Scientific, Fremont, California, USA) substrate staining system, and images were acquired by light microscopy (Olympus BX41TF, Olympus Corporation, Tokyo, Japan).

**Figure Legends**

**Supplementary Figure 1. Representative immunohistochemical staining pictures of CYP2E1 expression and mRNA expression of inflammatory factors in liver tissue among groups.**

(A) mRNA expression of IL-6 and MCP-1. (B) Immunohistochemical staining for CYP2E1 (400× original magnification) (C) Positive area of CYP2E1 staining, relative ratio of positive cells in the liver were analyzed with Image Pro-Plus 6.0. Data were presented as means±SEM. Pair-fed group; EtOH-fed: ethanol-fed group; EtOH-fed +QGHXR: QingganHuoxue Recipe group with ethanol-fed; EtOH-fed+QGR: Qinggan Recipe group with ethanol-fed; EtOH-fed+ HXR: Huoxue Recipe group with ethanol-fed. Compared with pair-fed group, *, *p*<0.05, **, *p*<0.01 ***, *p*<0.001; Compared with EtOH-fed group, #, *p*<0.05, ##, *p*<0.01, ###, *p*<0.001.

**Supplementary Figure 2. Representative immunohistochemical staining pictures of LXR expression and the mRNA expression of fatty acid oxidation factors in liver tissue among groups.**

(A) Immunohistochemical staining for LXR (400× original magnification). (B) The mRNA expression of PPARα, CPT1 and SREBP1. (C) Positive area of LXR staining, relative ratio of positive cells in the liver were analyzed with Image Pro-Plus 6.0 software. Data were presented as means±SEM. Pair-fed group; EtOH-fed: ethanol-fed group; EtOH-fed +QGHXR: QingganHuoxue Recipe group with ethanol-fed; EtOH-fed+QGR: Qinggan Recipe group with ethanol-fed; EtOH-fed+ HXR: Huoxue Recipe group with ethanol-fed. Compared with pair-fed group, *, *p*<0.05, **, *p*<0.01 ***, *p*<0.001; Compared with EtOH-fed group, #, *p*<0.05, ##, *p*<0.01, ###, *p*<0.001.

**Supplementary Figure 3. Establishment of a liver injury cell model in vitro, the best simulation condition of ethanol and the best intervention condition of QGHXR medicated serum in cells.**

(A) Effect of ethanol on the viability of AML12 and HepG2 cell. (B) Effects of different concentrations of QGHXR on the viability of AML12 and HepG2 cell stimulated for 24, 48 and 72 hours. (C) Effects of different concentrations of QGHXR on cell viability of AML12 and HepG2 cell induced by ethanol (100 mM). Data were presented as means±SEM. Con: Normal control group, Ethanol: ethanol group (100 mM), Ethanol+QGHXR: Qinggan Huoxue Recipe group induced by ethanol; Ethanol+QGR: Qinggan Recipe group induced by ethanol; Ethanol+HXR: Huoxue Recipe group induced by ethanol. Quantification of mRNA and protein level expression was normalized to β-actin levels. Compared with normal control group, *, *p*<0.05, **, *p*<0.01 ***, *p*<0.001; Compared with ethanol group, #, *p*<0.05, ##, *p*<0.01, ###, *p*<0.001.


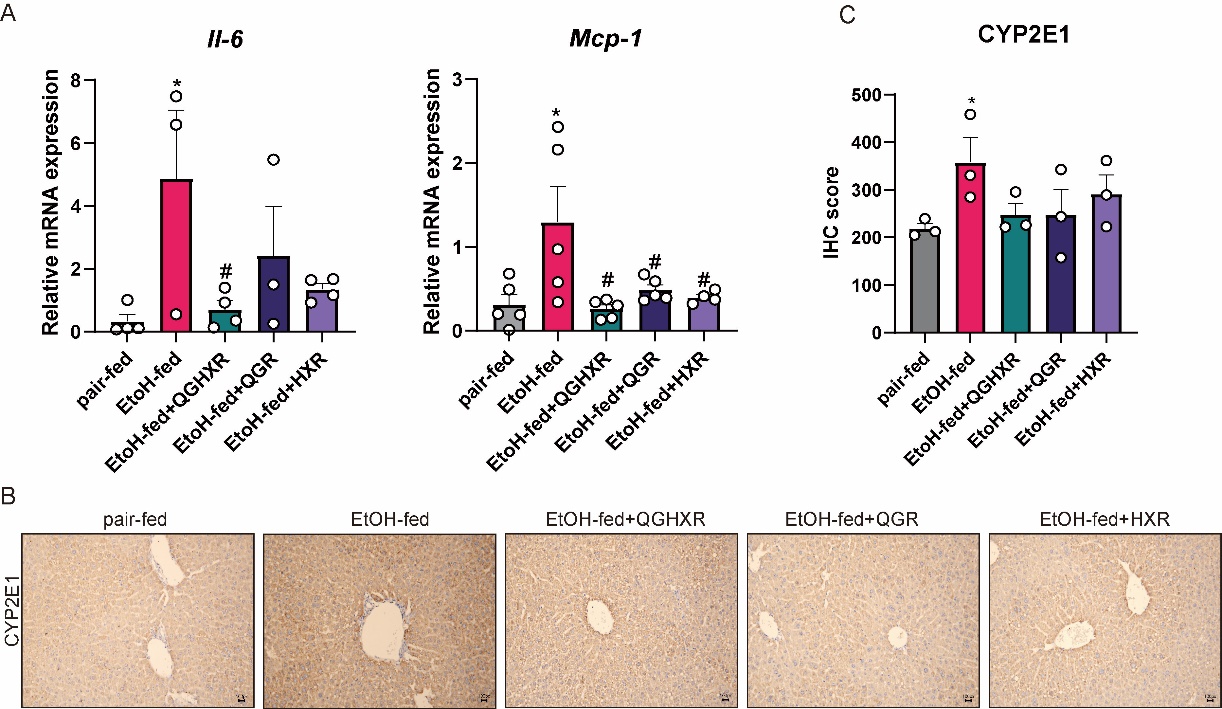


**Supplementary Figure 1. Representative immunohistochemical staining pictures of CYP2E1 expression and mRNA expression of inflammatory factors in liver tissue among groups.**

**
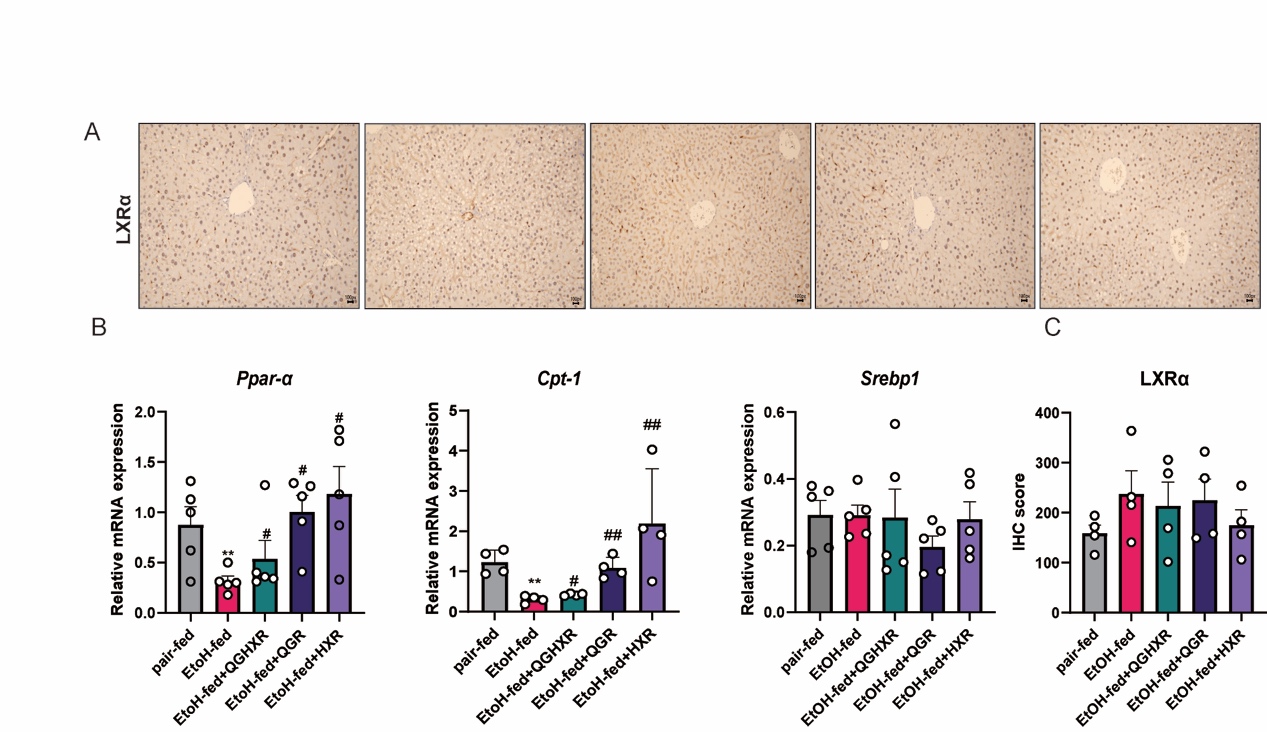
**

**Supplementary Figure 2. Representative immunohistochemical staining pictures of CYP2E1 expression and the mRNA expression of fatty acid oxidation factors in liver tissue among groups.**


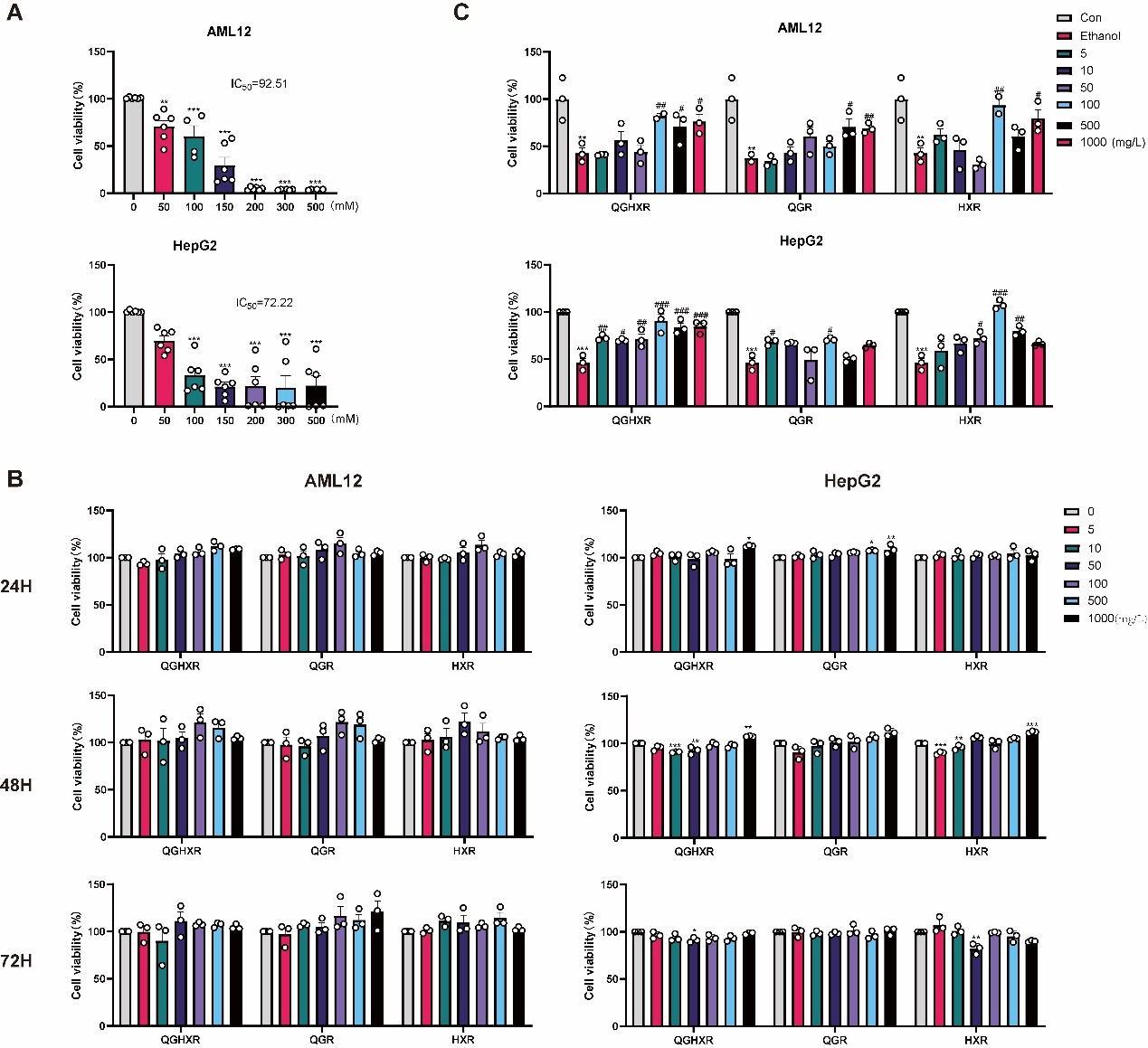


**Supplementary Figure 3.** Establishment of a liver injury cell model in vitro, the best simulation condition of ethanol and the best intervention condition of QGHXR, QGR, HXR medicated serum in cells.

**Supplementary Table 1.** Primers for real-time qPCR.

| **Genes** | **Sequence (5’to 3’)** | **Base pair（bp）** |  |
| --- | --- | --- | --- |
| *mActin* | mForward Primer 5’-GAGACCTTCAACACCCCAGC-3’ mReverse Primer 5’-ATGTCACGCACGATTTCCC-3’ | 263 |  |
| *mLxrα* | mForward Primer 5’-CCAAAATGCTGGGGAACG-3’  mReverse Primer 5’-GCGTGCTCCCTTGATGACA-3’ | 125 |  |
| *mLpcat3* | mForward Primer 5’-CCCCACATCACAGACGACTATC-3’  mReverse Primer 5’-TCTCACGGTCCCATTTTCATC-3’ | 192 |  |
| *mPerk* | mForward Primer 5’-GCTCAAAGACGAAAGCACAGAC-3’  mReverse Primer 5’-CCCACCGAGAAAGACCGAC-3’ | 153 |  |
| *mEif2α* | mForwardPrimer 5’-ACCTGGATACGGTGCCTACG-3’  mReverse Primer 5’-TCGAATTTTGACCGCTTGTG-3’ | 145 |  |
| *mAtf4* | mForward Primer 5’-ATGGAGCAAAACAAGACAGCA-3’  mReverse Primer 5’-TGCCTTACGGACCTCTTCTATC-3’ | 180 |  |
| *mChop* | mForward Primer 5’-AAACCTTCACTACTCTTGACCCTG-3’  mReverse Primer 5’-GGGCACTGACCACTCTGTTTC-3’ | 184 |  |
| *mAtf6* | mForward Primer 5’-AAAGTCCCAAGTCCAAAGCG-3’  mReverse Primer 5’-CTAGGTTTCACTCTTCGGGATTC-3’ | 116 |  |
| *mGrp78* | mForward Primer 5’-TTGTCCCCTTACACTTGGTATTG-3’  mReverse Primer 5’-TGTCTTTTGTTAGGGGTCGTTC-3’ | 170 |  |
| *mIre1α* | mForward Primer 5’-TTTGTTTTCCGAGGACAGTTTG-3’  mReverse Primer 5’-AGAAGTAGCGAAGCACGTTGG-3’ | 136 |  |
| *mXbp1* | mForward Primer 5’-CAAGTGGTGGATTTGGAAGAAG-3’  mReverse Primer 5’-TCCATTCCCAAGCGTGTTC-3’ | 119 |  |
| *mPparα* | mForward Primer 5’-CCTGAAAGATTCGGAAACTGC-3’ | 178 |  |
|  | mReverse Primer 5’-GACAAAAGGCGGGTTGTTG-3’ |  |  |
| *mSrebp1* | mForward Primer 5’- TCTGGAGACATCGCAAACAAG-3’ | 153 |  |
|  | mReverse Primer 5’-GGATGAGGTTCCAAAGCAGAC-3’ |  |  |
| *mCpt1* | mForward Primer 5’- GCCATACTGCTGTATCGTCGC-3’ | 116 |  |
|  | mReverse Primer 5’-CGGGAAGTATTGAAGAGTCGC-3’ |  |  |
| *mIl-6* | mForward Primer 5’- AAATGATGGATGCTACCAAACTG-3’ | 137 |  |
|  | mReverse Primer 5’- CTCTGGCTTTGTCTTTCTTGTTATC-3’ |  |  |
| *mTnf-α* | mForwardPrimer 5’- CCCTCCAGAAAAGACACCATG-3’ | 183 |  |
|  | mReverse Primer 5’- CACCCCGAAGTTCAGTAGACAG-3’ |  |  |
| *mMcp-1* | mForwardPrimer 5’- GCTGACCCCAAGAAGGAATG-3’ | 184 |  |
|  | mReverse Primer 5’- TTGAGGTGGTTGTGGAAAAGG-3’ |  |  |
| *hACTIN* | hForward Primer 5’- CTCCATCCTGGCCTCGCTGT-3’  hReverse Primer 5’- GCTGTCACCTTCACCGTTCC-3’ | 268 |  |
| *hLXRα* | hForwardPrimer 5’-GAAACTGAAGCGGCAAGAGG-3’  hReverse Primer 5’-AGCGCCGGTTACACTGTTG-3’ | 152 |  |
| *hLPCAT3* | hForwardPrimer 5’-GAAAAGGGCAAGGCAAAGTG-3’  hReverse Primer 5’- CCAGGCGTTGGTGTTGATG-3’ | 114 |  |
| *hPERK* | hForwardPrimer 5’-CTCGGGAAAAGGTAATGCG-3’  hReverse Primer 5’-ATCCATCTTTTCTTGCCACTTC-3’ | 119 |  |
| *hEIF2α* | hForward Primer 5’-GATTGAGGAAAAGAGGGGTGTG-3’  hReverse Primer 5’- TTTGGCTTCCATTTCTTCTGC-3’ | 154 |  |
| *hATF4* | hForward Primer 5’-CTCAGCACAGCCCCTCTACC-3’  hReverse Primer 5’- CCAGTTTCTCACCCTTTACTTTTG-3’ | 141 |  |
| *hCHOP* | hForward Primer 5’-AACCAGGAAACGGAAACAGAG-3’  hReverse Primer 5’- TTCACCATTCGGTCAATCAGA-3’ | 192 |  |
| *hATF6* | hForward Primer 5’-CTCAGCACAGCCCCTCTACC-3’  hReverse Primer 5’-CCAGTTTCTCACCCTTTACTTTTG-3’ | 125 | |
| *hGRP78* | hForward Primer 5’-GTCCTATGTCGCCTTCACTCC-3’  hReverse Primer 5’-GCACAGACGGGTCATTCCAC-3’ | 137 | |
| *hIRE1α* | hForward Primer 5’-GGAATTACTGGCTTCTGATAGGAC-3’  hReverse Primer 5’-GTGCGTTTTCTGAAGTCTGGTC-3’ | 177 | |
| *hXBP1* | hForward Primer 5’-ATGGATTCTGGCGGTATTGAC-3’  hReverse Primer 5’-GAGAAAGGGAGGCTGGTAAGG-3’ | 175 | |

Abbreviations: LXRα, Liver X receptor alpha; LPCAT3, Lysophosphatidylcholine acyltransferase 3; PERK, PKR-like endoplasmic reticulum kinase; eIF2α, Ukaryotic translation factor 2; ATF4, Activating transcription factor-4; CHOP, C/EBP homologous protein10; ATF6, Activating transcription factor-6; GRP78, Glucose-regulated protein 78; IRE1α, Inositol requiring enzyme1α; XBP1, X-box binding protein 1; CPT1, carnitine palmitoyltransferase 1;SREBP1, sterol regulatory element binding transcription factor 1; PPAR-α, Peroxisome proliferator-activated receptor-α.

Supplementary Table 2. Antibodies for Western blot.

| **Antibody** | **Number** | **Type** | **Company** | **Dilution ratio** |
| --- | --- | --- | --- | --- |
| Actin | R1207-1 | Rabbit | HUABIO,Hangzhou, China | 1:1000 |
| LXRα (for WB) | ab176323 | Rabbit | Abcam | 1:1000 |
| LXRα (for IHC) | ab41902 | Rabbit | Abcam | 1:50 |
| LPCAT3 | HAPL0516 | Rabbit | HUABIO,Hangzhou, China | 1:500 |
| phospho-PERK, p-PERK | 3179 | Rabbit | CST | 1:1000 |
| PERK | 20582-1-AP | Rabbit | Proteintech Group | 1:1000 |
| phospho-eIF2α, p-eIF2α | 3398S | Rabbit | CST | 1:1000 |
| Ukaryotic translation, eIF2α | 9722 | Rabbit | CST | 1:1000 |
| Activating transcription factor-4, ATF4 | 10835-1-AP | Rabbit | Proteintech Group | 1:500 |
| CHOP | 15204-1-AP | Rabbit | Proteintech Group | 1:200 |
| ATF6 | ab203119 | Rabbit | Abcam | 1:500 |
| GRP78 | 3177S | Rabbit | CST | 1:1000 |
| phospho-IRE1α, *p*-IRE1α | AF7150 | Rabbit | Affbiotech | 1:1000 |
| IRE1α | 3294S | Rabbit | CST | 1:1000 |
| XBP1u | ab220783 | Rabbit | Abcam | 1:1000 |
| CYP2E1 | 19937-1-AP | Rabbit | Proteintech | 1:50 |

Abbreviations: LXRα, Liver X receptor alpha; LPCAT3, Lysophosphatidylcholine acyltransferase 3; PERK, PKR-like endoplasmic reticulum kinase; eIF2α, Ukaryotic translation factor 2; ATF4, Activating transcription factor-4; CHOP, C/EBP homologous protein10; ATF6, Activating transcription factor-6; GRP78, Glucose-regulated protein 78; IRE1α, Inositol requiring enzyme1α; XBP1, X-box binding protein 1; CYP2E1, Cytochromes P450 2E1.
